# A Retrospective Study on the Association of the Systemic Inflammatory Response Index in Predicting the Severity of Obstructive Sleep Apnea

**DOI:** 10.3390/diagnostics15162091

**Published:** 2025-08-20

**Authors:** Shamnaz Shahul, Sindaghatta Krishnarao Chaya, Sana Khader Mathamveed, Komarla Sundararaja Lokesh, Suhail Azam Khan, Aishwarya R. Aladakatti, Venkatesh Kumar, Vivek Vasanthan, Jayaraj Biligere Siddaiah, Padukudru Anand Mahesh

**Affiliations:** 1 Department of Respiratory Medicine, JSS Academy of Higher Education and Research, Mysuru 570 015, India; shamnazshahul994@gmail.com (S.S.); chaya.sindaghatta@gmail.com (S.K.C.); lokeshpulmo@gmail.com (K.S.L.); khan2201@gmail.com (S.A.K.); aishwaryaaladakatti@gmail.com (A.R.A.); venkateshseri222@gmail.com (V.K.); vivekvasanthan0@gmail.com (V.V.); drjayarajbs@yahoo.com (J.B.S.); 2 Department of Community Medicine, JSS Academy of Higher Education and Research, Mysuru 570 015, India; sanakhadermathamveed@gmail.com

**Keywords:** systemic inflammatory response index, obstructive sleep apnea, apnea–hypopnea index, systemic inflammation, retrospective study

## Abstract

**Background/Objectives:** Obstructive sleep apnea (OSA) is a common disorder characterized by intermittent hypoxia and sleep fragmentation, assessed using the Apnea–Hypopnea Index (AHI). Systemic inflammation is central to OSA progression, and the systemic inflammatory response index (SIRI) has emerged as a potential biomarker for inflammatory diseases. This study investigates the relationship between SIRI and OSA severity while comparing other inflammatory markers. **Methods:** A retrospective study was conducted among 150 OSA patients at a tertiary care hospital. Based on AHI, patients were categorized into mild, moderate, and severe OSA groups. Blood parameters, including neutrophil, monocyte, and lymphocyte counts, were analyzed, and inflammatory indices (SIRI, NLR, PLR) were calculated. Correlation, ROCs, and regression analyses assessed associations between inflammatory markers and OSA severity. **Results:** SIRI demonstrated an excellent predictive ability for severe OSA with an AUC of 0.960 (cut-off: 1.105; sensitivity: 92.2%; specificity: 91.4%). The STOP-BANG score alone had lower discriminatory power (AUC: 0.737), but combining it with SIRI improved accuracy (AUC: 0.983). The best performance was observed when SIRI, STOP-BANG, PLR, and CRP were combined, yielding an AUC of 1.00, indicating perfect discrimination. **Conclusions:** SIRI shows strong predictive value for identifying severe OSA, underscoring its utility as a simple, cost-effective biomarker to aid early recognition and referral, particularly in primary care and resource-limited settings.

## 1. Introduction

Obstructive sleep apnea (OSA) is a highly prevalent yet underdiagnosed sleep disorder characterized by recurrent episodes of upper airway obstruction, leading to intermittent hypoxia, sleep fragmentation, and systemic inflammation [1,2]. Accumulating evidence suggests that chronic low-grade inflammation plays a pivotal role in OSA-associated complications, including cardiovascular disease, metabolic dysfunction, and endothelial impairment [3,4]. Intermittent hypoxia in OSA triggers a cascade of inflammatory responses, including oxidative stress, immune cell activation, and cytokine release, all of which contribute to disease progression [5,6,7]. Consequently, identifying reliable inflammatory biomarkers that correlate with OSA severity could enhance risk stratification, guide therapeutic decisions, and improve patient outcomes [8,9,10]. While several inflammatory indices, such as C-reactive protein (CRP), the neutrophil-to-lymphocyte ratio (NLR), and the platelet-to-lymphocyte ratio (PLR), have been investigated in OSA, their clinical utility remains inconsistent across studies [11,12,13,14].

The systemic inflammatory response index (SIRI) is an emerging biomarker that integrates neutrophil, monocyte, and lymphocyte counts to provide a composite measure of systemic inflammation [1,15]. Initially developed as a prognostic marker in oncology and cardiovascular diseases [4,15,16,17], SIRI has demonstrated superior predictive value over traditional inflammatory markers in various inflammatory conditions [1,15]. However, its role in OSA has not been well established, and it has yet to be systematically compared with other inflammatory indices in this context [9].

This study aims to investigate the association between SIRI and OSA severity and to compare its predictive performance with conventional inflammatory markers such as CRP, NLR, PLR, ESR, and monocyte counts [11,12,13,18]. Using correlation analysis, multivariable linear and logistic regression, and ROC curve evaluation, we will assess whether SIRI alone or in combination with clinical tools like the STOP-BANG score offers improved discrimination of OSA severity. These analyses are intended to explore the potential utility of SIRI as an accessible biomarker for risk stratification in patients with OSA.

## 2. Materials and Methods

### 2.1. Study Design and Setting

This retrospective observational study was performed over a six-month duration at JSS Hospital, Mysuru, a tertiary care facility in South India. The Institutional Ethics Committee approved the study (approval number: JSS/MC/IEC-23122024/04 NCT/2024-25), and all procedures adhered to the ethical norms established in the Declaration of Helsinki. The study included all consecutive adult patients diagnosed with obstructive sleep apnea (OSA) who underwent overnight polysomnography at the hospital’s sleep laboratory. Polysomnography was performed using the Philips Respironics Alice 6 G3 system with Sleepware G3 software version 3.8.1. The severity of OSA was classified based on the Apnea–Hypopnea Index (AHI), as per the American Academy of Sleep Medicine (AASM) 2012 criteria (AASM) with thresholds defining mild (AHI: 5–15 events/h), moderate (AHI: 15–30 events/h), and severe OSA (AHI > 30 events/h).

### 2.2. Inclusion and Exclusion Criteria

**Figure 1:**
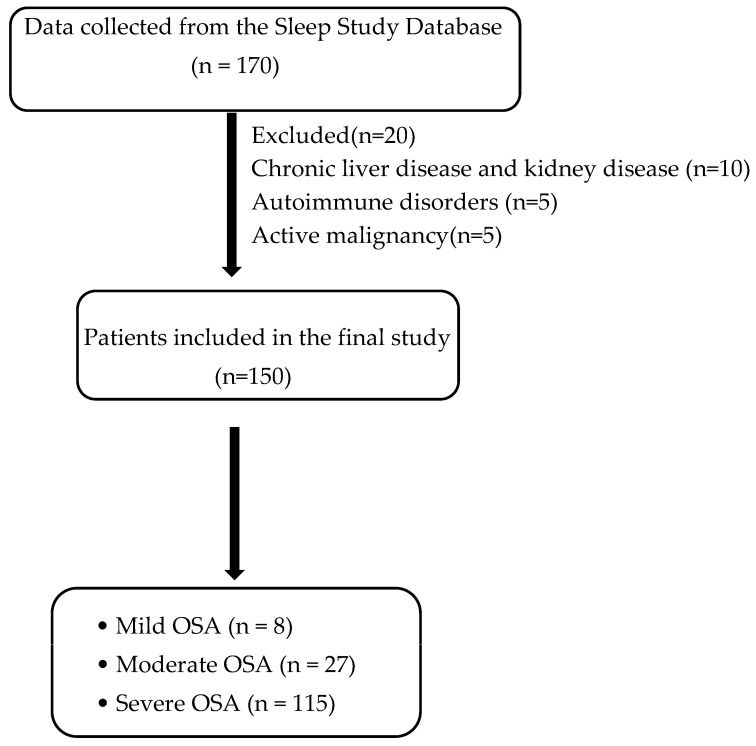
Flow diagram of patient selection criteria.

Eligible participants were adults aged 18 years or older with a confirmed diagnosis of OSA. Patients with hepatic or renal dysfunction, active malignancies, or autoimmune disorders were excluded to minimize potential confounding effects on inflammatory marker levels (Figure 1).

### 2.3. Data Collection and Biomarker Analysis

Demographic and clinical characteristics, including age, gender, body mass index (BMI), and comorbid conditions, were systematically recorded. Polysomnography reports were analyzed, and sleep data was collected and systematically recorded. Oxygen saturation (SpO_2_) was continuously recorded during overnight polysomnography using standard pulse oximetry at a sampling rate of 1 Hz. For each patient, the lowest (nadir) SpO_2_ and the cumulative time spent below 90% saturation were extracted from the recording. The oxygen desaturation index (ODI) was not consistently available in the sleep study reports and was, therefore, not included in the analysis.

Blood samples were collected at the time of diagnosis to assess inflammatory markers, including complete blood count parameters (neutrophil, lymphocyte, monocyte, and platelet counts) and biochemical markers such as C-reactive protein (CRP) and erythrocyte sedimentation rate (ESR).

To assess systemic inflammation in patients with OSA, several inflammatory indices were calculated. The systemic inflammatory response index (SIRI) was determined using the following formula: (Neutrophils × Monocytes)/Lymphocytes, providing a composite measure of immune activation. Additionally, the neutrophil-to-lymphocyte ratio (NLR) was derived by dividing the neutrophil count by the lymphocyte count, reflecting the balance between innate and adaptive immune responses. Similarly, the platelet-to-lymphocyte ratio (PLR) was calculated by dividing the platelet count by the lymphocyte count, serving as an indicator of platelet-driven inflammation. These indices were analyzed to explore their association with OSA severity and compare their predictive value in assessing systemic inflammation in affected individuals.

### 2.4. Statistical Analysis

Data analysis was performed using IBM SPSS Statistics for Windows, version 25.0 (IBM Corp., Armonk, NY, USA). Continuous variables were assessed for normality using the Kolmogorov–Smirnov test. Normally distributed variables were presented as means ± standard deviations (SDs), while non-normally distributed variables were expressed as medians (interquartile ranges, IQRs). Categorical variables were reported as frequencies and percentages. Comparisons between OSA severity groups (mild, moderate, and severe) were conducted using the one-way analysis of variance (ANOVA) for normally distributed continuous variables and the Kruskal–Wallis test for non-normally distributed variables. The chi-square test was employed to assess differences in categorical variables across groups.

To evaluate associations between AHI and inflammatory markers (SIRI, NLR, PLR, CRP, and ESR), Pearson’s correlation coefficient was used for normally distributed markers, while Spearman’s rank correlation was applied for non-normally distributed variables. A multivariate logistic regression analysis was conducted to identify independent predictors of moderate-to-severe OSA (AHI ≥ 15 events/h). Variables with *p* < 0.10 in univariate analysis were included in the regression model using a stepwise selection approach. Adjusted odds ratios (ORs) with 95% confidence intervals (CIs) were reported to quantify the strength of associations. A two-tailed *p*-value < 0.05 was considered statistically significant for all analyses.

An ROC analysis was performed to determine the discriminative capacity of the variables, AUC values were computed with 95% confidence intervals, and optimal cut-off points were identified using Youden’s index.

## 3. Results

### 3.1. Baseline Characteristics

The study analyzed 150 patients diagnosed with obstructive sleep apnea (OSA). The demographic and clinical characteristics of the participants are summarized in Table 1 and Table 2. The mean age of the patients was 52.84 ± 13.02 years, and 55.3% were male. The prevalence of comorbidities was as follows: hypertension (30%), diabetes mellitus (2.1%), both diabetic and hypertensive (21.3%), and obesity (mean BMI: 33.22 ± 6.05 kg/m^2^). Of the total study population, 78 patients had a neck circumference of >40 cm (52%), and 72 people had a neck circumference <40 cm (48%). Based on the Apnea–Hypopnea Index (AHI), the distribution of patients was as follows: mild OSA, 7 patients (4.7%); moderate OSA, 28 patients (18.7%); and severe OSA, 115 patients (76.7%). Patients with severe OSA had significantly higher mean BMIs and a greater prevalence of hypertension and diabetes mellitus compared to those with mild or moderate OSA (*p* < 0.05); see Table 3.

### 3.2. Comparisons of Inflammatory Markers Across OSA Severity Groups

A comparative analysis of inflammatory markers, lipid profiles, and oxygen saturation across mild, moderate, and severe OSA groups revealed significant differences in several parameters; see Table 4. SIRI, monocyte count, PLR, ESR, CRP, and NLR levels were progressively higher in severe OSA cases compared to mild and moderate cases (*p* < 0.05). These findings highlight the systemic inflammatory burden associated with worsening OSA.

Total cholesterol (TC), LDL cholesterol, and triglyceride (TG) levels were significantly elevated in severe OSA patients (*p* < 0.05), suggesting a potential link between OSA-related inflammation and dyslipidemia. In contrast, HDL cholesterol levels did not significantly differ across groups (*p* = 0.811).

A significant inverse correlation was observed between the lowest oxygen saturation (SPO2) and inflammatory markers, with lower SPO2 levels in severe OSA correlating with higher SIRI, monocyte counts, and CRP levels (*r* = −0.38, *p* < 0.001).

The cumulative time spent with oxygen saturation below 90% increased markedly with OSA severity, highlighting the progressive nature of nocturnal hypoxemia. Patients with mild OSA spent an average of 5.53 ± 3.6 min below this threshold, which rose sharply to 68.60 ± 32 min in moderate OSA and escalated to 226.2 ± 85 min in severe cases (*p* < 0.001). This desaturation burden showed a strong positive correlation with AHI (*r* = 0.403, *p* < 0.001). Neck circumference also demonstrated a significant correlation with AHI (*r* = 0.404, *p* < 0.001), and STOP-BANG scores were significantly associated with AHI (*r* = 0.347, *p* < 0.001). In the correlation analysis, the STOP BANG score revealed a significant positive correlation with AHI (*r* = 0.347, *p* < 0.001), indicating an increased severity of OSA in people with higher STOP BANG scores (Table 5).

### 3.3. Correlation Between AHI and Inflammatory Markers

**Figure 2:**
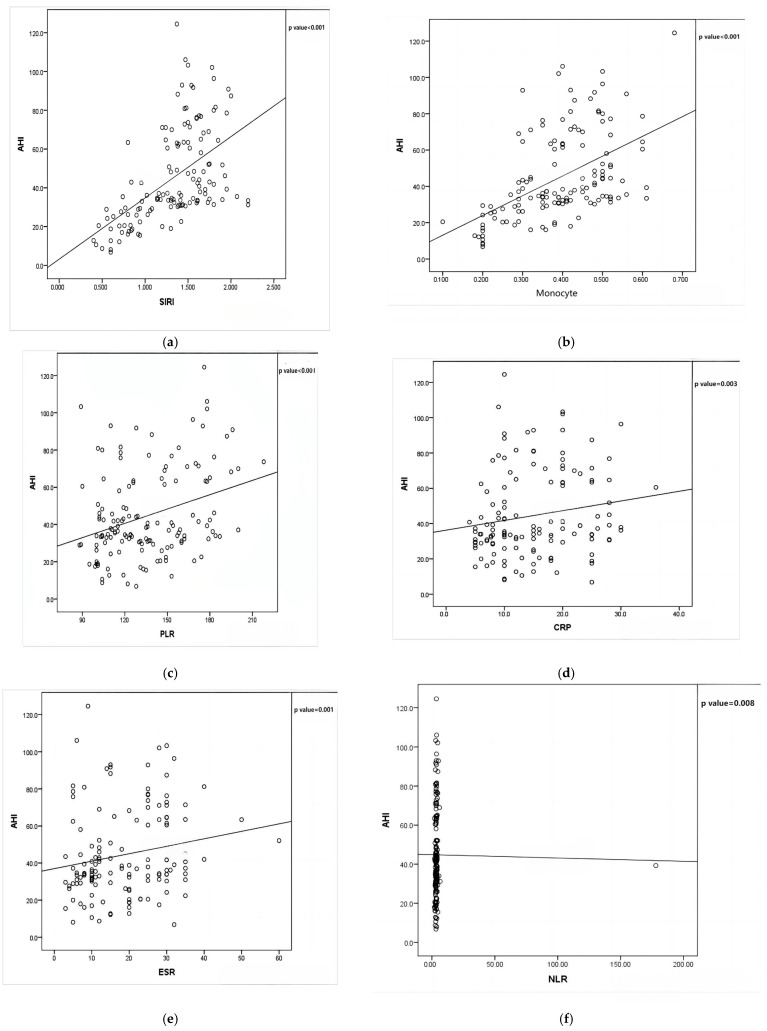
Scattered plots showing correlation of inflammatory markers and AHI. (**a**) Scattered plot between AHI and SIRI. (**b**) Scattered plot between AHI and monocyte. (**c**) Scattered plot between AHI and PLR. (**d**) Scattered plot between AHI and CRP. (**e**) Scattered plot between AHI and ESR. (**f**) Scattered plot between AHI and NLR.

**Figure 3:**
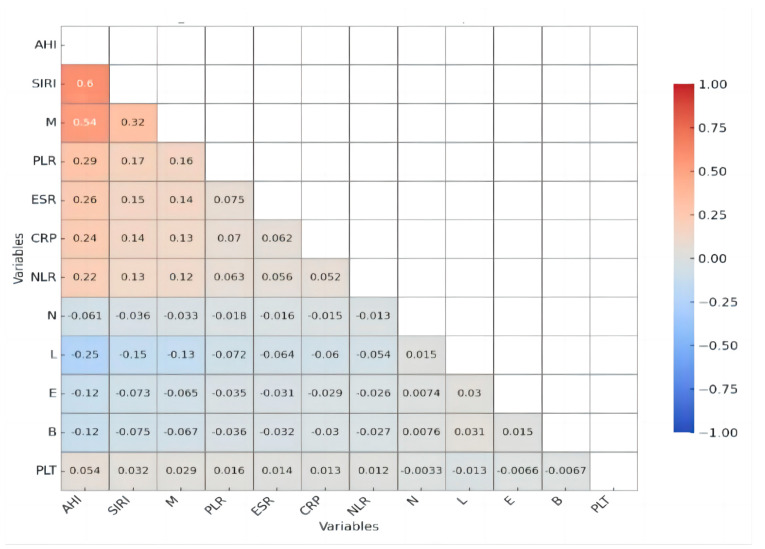
Correlation matrix heat map of inflammatory parameters and AHI.

A Pearson correlation analysis demonstrated significant associations between AHI and multiple inflammatory markers, underscoring the role of systemic inflammation in obstructive sleep apnea (OSA) severity. Notably, SIRI exhibited a moderate positive correlation with AHI (*r* = 0.597, *p* < 0.001), making it the most strongly associated inflammatory marker. The monocyte count (M) also showed a moderate positive correlation with AHI (*r* = 0.536, *p* < 0.001). Among other inflammatory markers, CRP displayed a weak but significant positive correlation with AHI (*r* = 0.241, *p* = 0.003), suggesting a role for systemic inflammation, though less pronounced. Similarly, PLR (*r* = 0.290, *p* < 0.001), ESR (*r* = 0.257, *p* = 0.001), and NLR (*r* = 0.216, *p* = 0.008) all showed weak yet significant associations with AHI, indicating that, while these markers may contribute to the inflammatory profile of OSA, their predictive value may be lower than that of SIRI and monocyte counts. A correlation analysis treated AHI as a continuous variable for comparative assessment (Figure 2 and Figure 3).

### 3.4. Inflammatory Markers and OSA Severity

**Figure 4:**
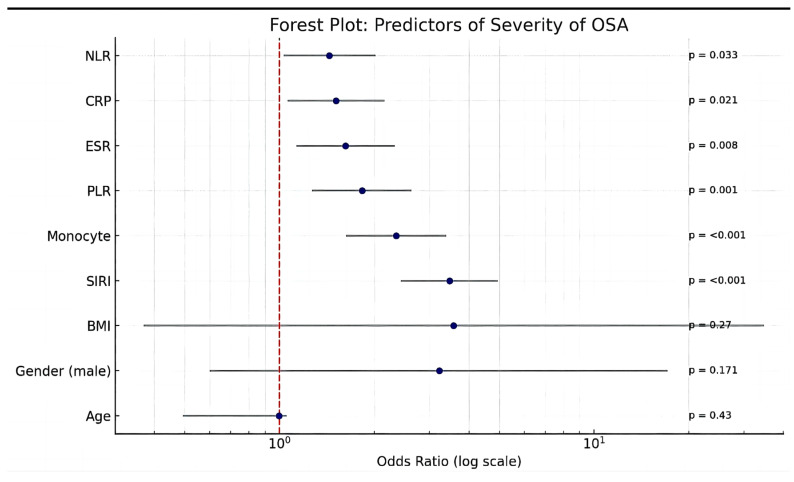
Forest plot showing predictors of severity of OSA. The dashed line represents no risk. Any variable whose 95% CI lies on the right side of the dashed line is a significant risk factor and to the left is protective. If the 95% CI crosses the dashed line, it is neither a significant risk or protective factor.

A multivariate logistic regression analysis identified several key inflammatory markers as significant independent predictors of obstructive sleep apnea (OSA) severity (Figure 4). The systemic inflammatory response index (SIRI) was the strongest predictor of severe OSA, with an odds ratio (OR) of 3.47 (95% CI: 2.43–4.95, *p* < 0.001), indicating a threefold increase in the odds of severe OSA per unit increase in SIRI.

Monocyte counts were also strongly associated with OSA severity, with an OR of 2.35 (95% CI: 1.63–3.39, *p* < 0.001), suggesting that monocyte-driven immune activation plays a crucial role in disease progression. Similarly, an elevated platelet-to-lymphocyte ratio (PLR) was significantly linked to increased OSA severity (OR: 1.83, 95% CI: 1.27–2.63, *p* = 0.001), reinforcing the role of platelet-driven inflammation and thrombosis in the pathophysiology of OSA. Markers of systemic inflammation, including raised erythrocyte sedimentation rate (ESR) (OR: 1.62, 95% CI: 1.13–2.32, *p* = 0.008) and C-reactive protein (CRP) (OR: 1.51, 95% CI: 1.06–2.15, *p* = 0.021), as well as an elevated neutrophil-to-lymphocyte ratio (NLR) (OR: 1.44, 95% CI: 1.03–2.02, *p* = 0.033), were associated with OSA severity. Age was not a significant predictor of OSA severity (OR: 0.995, 95% CI: 0.492–1.053, *p* = 0.43). Even though male gender and BMI exhibited a 3-fold rise in the odds of severe OSA per unit increase, both were not significant.

Linear regression analysis for identifying predictors of severe OSA revealed SIRI, monocytes, and PLR as significant independent predictors of obstructive sleep apnea (OSA) severity after adjusting for age, sex, BMI, and lipid parameters; see Table 6.

Incorporating AGE, BMI, SIRI, total cholesterol, LDL, triglycerides, CCI (Charlson’s comorbidity index), and sex (male), the model exhibited an R^2^ value of 0.357, signifying that 35.7% of the variance in AHI is elucidated through the independent variables. The regression model demonstrated statistical significance (F = 9.231, *p* < 0.001). SIRI (β = 25.848, *p* < 0.001) exhibited a robust positive correlation with AHI, indicating that elevated SIRI levels substantially enhance AHI. The correlation between monocyte levels and AHI yielded an R^2^ value of 0.348, accounting for 34.8% of the variance in AHI (F = 8.862, *p* < 0.001) and indicating a robust, significant positive association with AHI (β = 90.391, *p* < 0.001). PLR and AHI had R^2^ value of 0.264, accounting for 26.4% of the variation in AHI (F = 5.975, *p* < 0.001), (β = 0.201, *p* = 0.002). The correlation between CRP and AHI yielded an R^2^ of 0.210, accounting for 21.0% of the variation in AHI (F = 4.413, *p* < 0.001). However, CRP did not exhibit a significant association with AHI (β = 0.144, *p* = 0.616) following the adjustment for confounding variables. A similar observation was noted with ESR and AHI, yielding an R^2^ of 0.221, which accounts for 22.1% of the variation in AHI (F = 4.719, *p* < 0.001), although it lacks statistical significance (β = 0.266, *p* = 0.141) after confounding variables are controlled for. Even though NLR and AHI exhibited an R^2^ of 0.223, accounting for 22.3% of the variation in AHI (F = 4.779, *p* < 0.001), it was not statistically correlated with AHI (β = 4.225, *p* = 0.111) after confounding factors were adjusted for.

In this model of analysis, BMI and male gender were consistently significant predictors of AHI across all models. Among inflammatory markers, SIRI and monocyte demonstrated the strongest positive associations, and PLR exhibited a moderate positive association with AHI, respectively, after adjusting for the confounding factors (age, gender, BMI, lipid parameters, and CCI), whereas CRP, ESR, and NLR were not significant predictors. These findings suggest that metabolic and inflammatory markers may play a crucial role in predicting AHI, independent of traditional risk factors.

**Figure 5:**
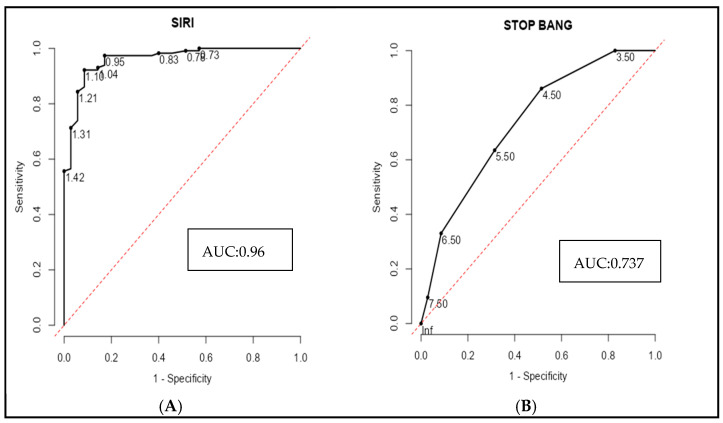

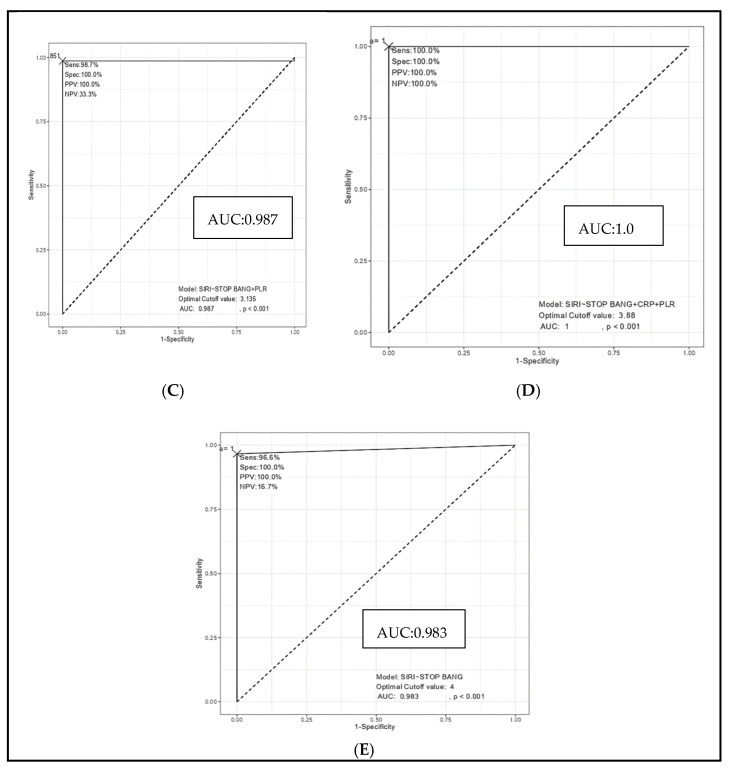
ROC curves comparing the predictive accuracy of SIRI, STOP-BANG, and combined biomarker models for severe OSA.

A receiver operating characteristic (ROC) curve analysis was performed to evaluate the ability of inflammatory markers and the STOP-BANG score to discriminate severe OSA from mild and moderate cases. Among individual biomarkers, the systemic inflammatory response index (SIRI) demonstrated excellent diagnostic accuracy, with an area under the curve (AUC) of 0.961, sensitivity of 92.2%, and specificity of 91.4% at a cut-off value of 1.105 (Figure 5A). In contrast, the STOP-BANG score alone exhibited more modest discrimination (AUC = 0.737), with a sensitivity of 86.1% but a lower specificity of 48.6% at a cut-off of 4.5 (Figure 5B). Combining SIRI with the STOP-BANG score significantly enhanced the diagnostic performance (AUC = 0.983), yielding both high sensitivity (96.6%) and perfect specificity (100%) (Figure 5E). This performance was further improved by incorporating the platelet-to-lymphocyte ratio (PLR), resulting in an AUC of 0.987. (Figure 5C). Notably, the combined model including SIRI, STOP-BANG scores, PLR, and CRP achieved perfect diagnostic accuracy (AUC = 1.0), with 100% sensitivity, specificity, and predictive values across the board (Figure 5D). These findings underscore the incremental value of integrating systemic inflammatory markers with established clinical risk scores to improve the identification of patients with severe OSA (Figure 5 and Table 7).

## 4. Discussion

The present study provides important evidence linking systemic inflammation to the severity of obstructive sleep apnea (OSA). Our findings demonstrate that a range of inflammatory markers are not only significantly elevated in individuals with more severe OSA but also independently associated with the apnea–hypopnea index (AHI), a key clinical measure of disease burden. Among these, the systemic inflammatory response index (SIRI) emerged as the most robust correlate (*r* = 0.597, *p* < 0.001) and a powerful independent predictor of OSA severity (OR: 3.47, 95% CI: 2.43–4.95, *p* < 0.001). Monocyte counts (*r* = 0.536, *p* < 0.001) and PLR also showed strong and consistent associations, reinforcing the central role of innate immune activation. Additional markers, such as ESR, CRP, and NLR, exhibited statistically significant but comparatively weaker associations with AHI, suggesting a broader inflammatory profile in OSA. Importantly, multivariate logistic regression and adjusted linear regression analyses consistently identified SIRI, monocyte counts, and PLR as independent predictors of OSA severity, even after key confounders such as age, gender, and BMI were accounted for [9,19].

Among the inflammatory indices evaluated, the systemic inflammatory response index (SIRI), a composite biomarker calculated from neutrophil, monocyte, and lymphocyte counts, demonstrated the strongest association with OSA severity in our cohort [1]. SIRI, as a novel inflammatory marker, provides a more comprehensive assessment of systemic inflammation compared to individual biomarkers. By incorporating neutrophil, monocyte, and lymphocyte counts into a single index, SIRI captures multiple aspects of immune activation, offering a potentially superior measure of inflammation in OSA compared to traditional markers such as CRP or NLR. This finding reinforces the concept that chronic, low-grade systemic inflammation plays a central role in the pathophysiological cascade of OSA [4,18,20]. While SIRI has been extensively studied as a prognostic marker in cardiovascular diseases, metabolic syndrome, and various malignancies, its application in the context of OSA has been limited and underexplored. A small number of recent investigations have suggested that elevated SIRI levels may reflect the systemic inflammatory milieu in OSA, yet robust, large-scale evidence has remained scarce [1,21]. Our results not only corroborate this emerging association but also position SIRI as a particularly sensitive and independent predictor of OSA severity, outperforming other commonly used markers such as CRP and NLR. Given its derivation from routine hematological parameters, SIRI offers a practical, cost-effective, and scalable tool for clinical assessment [10,22]. Variability across previous studies may be attributable to differences in sample size, population characteristics, AHI thresholds, or laboratory measurement protocols. Nonetheless, our findings provide compelling support for the incorporation of SIRI into future risk stratification frameworks for OSA [13,23].

Our study also identified peripheral blood monocyte counts as a robust and independent inflammatory marker associated with OSA severity [3]. Monocytes are key effectors of the innate immune response and play a pivotal role in orchestrating chronic inflammation—a hallmark of OSA-related pathophysiology. Recurrent episodes of intermittent hypoxia (IH), a defining feature of OSA, are known to activate monocyte proliferation and recruitment, both in bone marrow and peripheral circulation [24,25]. These activated monocytes, and their differentiated forms as tissue macrophages, contribute to vascular inflammation, endothelial dysfunction, and oxidative stress by releasing pro-inflammatory cytokines and reactive oxygen and nitrogen species [6,26]. Histopathological studies of chronic inflammatory states, including in OSA, have shown an increased infiltration of mononuclear cells, fibroblast activation, and tissue remodeling, all of which may be driven in part by elevated monocyte activity. The strong positive correlation between monocyte counts and AHI in our dataset is consistent with the emerging literature that implicates monocytes as key mediators of systemic inflammation in OSA [3,15,27].

Our study demonstrated a significant association between the platelet-to-lymphocyte ratio (PLR) and OSA severity, with higher PLR values observed in individuals with more severe disease. PLR has gained recognition as a composite marker of systemic inflammation [5,28,29], reflecting the interplay between platelet activation and lymphocyte suppression. Platelets, beyond their traditional role in hemostasis, are now understood to be active mediators in inflammatory and immune responses [28]. They release a wide array of cytokines and chemokines, and their increased aggregation and activation in inflammatory states can contribute to vascular injury and oxidative stress—mechanisms highly relevant in the pathogenesis of OSA [30,31,32]. Concurrent lymphopenia, often observed in chronic inflammatory conditions, may signify immune exhaustion or dysregulation. Thus, an elevated PLR may capture both the heightened platelet reactivity and impaired adaptive immunity seen in OSA. Previous studies have reported similar trends, linking PLR to both OSA severity and cardiovascular risk in this population [5,7,33]. However, the strength of these associations has varied, likely due to heterogeneity in study design, population characteristics, and adjustment for confounders such as obesity, insulin resistance, and dyslipidemia. Our findings reinforce the potential of PLR as an accessible inflammatory biomarker that may reflect both disease severity and systemic immune imbalance in OSA.

In addition to SIRI, monocyte counts, and PLR, our study identified the neutrophil-to-lymphocyte ratio (NLR), erythrocyte sedimentation rate (ESR), and C-reactive protein (CRP) as independent inflammatory markers associated with OSA severity. NLR, a widely used indicator of systemic inflammation, reflects the balance between neutrophil-driven innate immune activation and lymphocyte-mediated adaptive immune regulation [20,32]. Elevated NLR has been linked to oxidative stress, endothelial dysfunction, and hypoxia-induced inflammation—all key mechanisms implicated in OSA pathophysiology [12,16,34,35]. ESR and CRP, classical acute-phase reactants, also showed a graded increase with increasing OSA severity in our cohort [36,37]. CRP, in particular, is a sensitive marker of low-grade systemic inflammation and has been consistently associated with cardiovascular morbidity in OSA [11]. ESR, though less specific, mirrors chronic inflammatory activity and is influenced by cytokine-mediated alterations in plasma protein composition [8]. The independent associations of these markers with the apnea–hypopnea index (AHI), even after adjusting for age, gender, and BMI, suggest that systemic inflammation in OSA is not merely a by-product of comorbid conditions but is an intrinsic component of disease severity. These findings align with prior studies but add robustness by incorporating multivariate analysis in a well-characterized cohort. Collectively, NLR, ESR, and CRP complement more novel indices like SIRI and PLR in elucidating the inflammatory milieu of OSA and may offer additional value in clinical phenotyping and risk assessment.

The observed associations between systemic inflammatory markers and OSA severity likely reflect underlying pathophysiological mechanisms driven by intermittent hypoxia (IH) and sleep fragmentation [38,39]. IH, a hallmark of OSA, triggers repetitive cycles of hypoxia and reoxygenation that promote oxidative stress, sympathetic nervous system activation, and the upregulation of pro-inflammatory transcription factors such as NF-κB [13,14,40,41]. This cascade stimulates the production of cytokines (e.g., IL-6, TNF-α), acute-phase proteins (e.g., CRP), and adhesion molecules, which collectively contribute to endothelial dysfunction and systemic inflammation [2,8,42]. Additionally, IH induces monocytopoiesis in the bone marrow and mobilization of activated monocytes and neutrophils into the circulation, which may explain the elevated monocyte count, NLR, and SIRI observed in this study [24,25,43]. Sleep fragmentation further exacerbates this inflammatory response by altering hypothalamic–pituitary–adrenal axis function and impairing immune regulation [39,44]. These inflammatory processes are not confined to the respiratory system but have systemic effects, contributing to the heightened cardiovascular and metabolic risk in OSA patients [17,41,45]. This inflammatory cascade not only exacerbates OSA severity but also increases the risk of comorbidities such as hypertension, cardiovascular disease, and metabolic disorders [17,45,46]. Thus, the inflammatory profile observed in OSA is not merely a consequence of comorbidities but is intrinsically tied to the disease pathogenesis, offering potential targets for risk stratification and therapeutic intervention [46].

Among all inflammatory markers analyzed in this study, SIRI showed the strongest correlation with AHI, suggesting that it may be a superior marker for assessing OSA-related inflammation [1]. Topuz et al. reported similar findings, demonstrating that SIRI was significantly elevated in OSA patients and was associated with cardiovascular risk factors. Unlike other inflammatory markers that may be influenced by transient infections or acute inflammation, SIRI provides a more stable representation of chronic immune dysregulation. Given its strong correlation with OSA severity, SIRI may function as a significant biomarker for the identification of high-risk patients and for the selection for therapeutic measures [1]. Elevated systemic inflammatory markers, particularly SIRI, could help identify OSA patients at an increased risk of cardiovascular and metabolic complications, thereby guiding earlier initiation of therapeutic interventions such as CPAP therapy.

The identification of systemic inflammatory markers such as SIRI, monocyte counts, PLR, NLR, ESR, and CRP as independent predictors of OSA severity has important clinical implications. These findings have important clinical implications for the early detection of severe OSA, particularly in resource-limited or primary care settings where access to polysomnography is constrained. The STOP-BANG score, while widely used for OSA screening due to its simplicity, demonstrated limited specificity for the discrimination of severe OSA in our cohort. However, when combined with SIRI, a readily available and inexpensive blood-based biomarker of systemic inflammation, the diagnostic precision of severe OSA was substantially enhanced. The addition of PLR and CRP further refined predictive accuracy, culminating in a model that perfectly discriminated severe OSA from less severe forms. This suggests that a composite panel incorporating inflammatory markers with clinical screening tools could be a powerful strategy for triaging high-risk individuals for definitive sleep studies, especially in primary care and resource-limited settings. Given the well-established association between severe OSA and cardiovascular morbidity and mortality, early identification through such an approach could facilitate timely intervention and reduce downstream complications [47]. While these results are promising, validation in prospective, multicenter cohorts is essential to confirm their generalizability and assess the feasibility of implementation in routine clinical practice. There is a need for future studies to evaluate whether monitoring inflammatory markers could provide insights into the treatment response in reducing systemic inflammation and long-term morbidity and mortality, particularly for interventions such as CPAP, weight loss, or anti-inflammatory therapies. As precision medicine continues to evolve in sleep medicine, these biomarkers hold promise for guiding individualized management strategies, improving prognostication, and potentially identifying novel therapeutic targets aimed at modulating the systemic inflammatory burden in OSA.

### Strengths and Limitations

Our study boasts several strengths. First, the relatively large sample size allowed for a robust statistical analysis and subgroup comparisons across varying severities of OSA. Second, the diagnosis of OSA was rigorously confirmed using Level 1 attended overnight polysomnography, the gold standard for sleep-disordered breathing, thereby ensuring high diagnostic accuracy. Third, we comprehensively evaluated a wide panel of inflammatory markers, including both traditional indices (e.g., CRP, ESR, and NLR) and novel composite markers such as SIRI and PLR, providing a nuanced view of the systemic inflammatory landscape in OSA. Fourth, the use of both correlation and multivariate regression models allowed us to adjust for key confounders and identify independent predictors of disease severity.

However, certain limitations should be acknowledged. The retrospective design of the study inherently limits the ability to establish causal relationships between inflammation and OSA severity. While significant associations were observed, prospective studies are necessary to determine whether inflammation actively contributes to disease progression or is merely a consequence of OSA-related physiological stress. Furthermore, the absence of longitudinal data restricts insights into the dynamic interplay between SIRI and OSA over time, preventing an evaluation of how inflammatory markers fluctuate with disease progression or therapeutic interventions. Additionally, potential confounders such as smoking status, alcohol consumption, medication use, and underlying cardiac comorbidities were not systematically accounted for, which could have influenced inflammatory marker levels. The study population consisted exclusively of patients with a confirmed diagnosis of OSA, with a predominance of severe cases, reflecting a tertiary care referral bias. As such, the findings may not be generalizable to undiagnosed or community-based populations. Finally, while we included the lowest SpO_2_ and time spent below 90% saturation, other measures of nocturnal hypoxemia, such as the oxygen desaturation index (ODI), were unavailable and could have provided additional granularity in assessing hypoxia–inflammation relationships. Future prospective studies should address these limitations by incorporating broader population samples, detailed lifestyle and clinical variables, and comprehensive nocturnal hypoxia metrics to better define the role of systemic inflammation in OSA severity and progression.

## 5. Conclusions

This study provides compelling evidence that systemic inflammation is a central feature of obstructive sleep apnea (OSA) and correlates strongly with disease severity. Among the biomarkers evaluated, the systemic inflammatory response index (SIRI) showed the strongest association with the apnea–hypopnea index (AHI) and emerged as the most powerful independent predictor of severe OSA. Monocyte counts, PLR, CRP, NLR, and ESR also demonstrated significant associations, reinforcing the involvement of both innate and adaptive immune responses in OSA pathophysiology.

Importantly, ROC analysis revealed that SIRI alone achieved excellent discriminatory performance, which was significantly enhanced when combined with the STOP-BANG score, PLR, and CRP, culminating in perfect diagnostic accuracy. This highlights the clinical potential of integrating inflammatory markers with standard screening tools for the early and accurate identification of patients at risk for severe OSA, particularly in resource-limited settings. These findings support a biomarker-guided approach to OSA risk stratification and underscore the need for prospective studies to validate these markers across diverse populations. Future research should also examine whether the effective treatment of OSA reduces systemic inflammation and mitigates downstream cardiovascular and metabolic risks.

## Figures and Tables

**Table 1:** Distribution of the participants based on their sociodemographic characteristics.

Variable	Frequency (*N*)	Percentage (%)
Age group		
Less than 25 years	4	2.7
25 to 40 years	25	16.7
40 to 60 years	72	48
Above 60 years	49	32.6
Gender		
Male	83	55.3
Female	67	44.7
BMI category		
Underweight	0	0
Normal	6	4
Overweight	42	28
Obese	102	68
Comorbidity status		
No comorbidities	49	32.6
DM	4	2.7
Hypertension	45	30
Hypothyroidism	7	4.6
DM/hypertension	32	21.3
DM/hypothyroidism	2	1.3
Hypertension/hypothyroidism	4	2.7
DM/hypertension/hypothyroidism	4	2.7
Hypertension/seizure	1	0.7
Hypertension/DM/hyperthyroid	1	0.7
Hypertension/hypothyroid/seizure	1	0.7
Charlson’s Comorbidity Index		
Low	89	59.3
Moderate	55	36.7
High	6	4
Neck circumference		
>40 cm	78	52
<40 cm	72	48

**Table 2:** Distribution of participants based on patient characteristics.

Variable	Frequency (*N*)	Percentage (%)
AHI severity		
Mild	7	4.7
Moderate	28	18.7
Severe	115	76.6
Anemia		
Present	44	29.3
Absent	106	70.7
High TSH		
Present	56	37.3
Absent	94	62.7
Total cholesterol high		
Present	150	100
High CRP		
Present	150	100
High ESR		
Present	85	56.7
Absent	65	43.3
PASP severity		
Mild	61	40.7
Moderate	76	50.6
severe	13	8.7
High PCO2 (ABG)		
Present	35	23.3
Absent	115	76.7
Low PO2 (ABG)		
Present	70	46.7
Absent	80	53.3

**Table 3:** Factors associated with severity of AHI (chi-square test).

Variable	AHI INDEX			Chi-Square/Fisher’s	*p*-Value
	Mild	Moderate	Severe		
Age group				5.593	0.470
Less than 25 years	0	1 (25)	3 (75)		
25 to 40 years	1 (4)	2 (8)	22 (88)		
40 to 60 years	2 (2.8)	13 (18.1)	57 (79.2)		
Above 60 years	4 (8.2)	12 (24.5)	33 (67.3)		
Gender				10.584	0.005
Male	2 (2.4)	9 (10.8)	72 (86.7)		
Female	5 (7.5)	19 (28.4)	43 (64.2)		
BMI category		0		34.096	0.014
Underweight	0	4 (66.7)	0		
Normal	4 (9.5)	7 (16.7)	2 (33.3)		
Overweight	3 (2.9)	17 (16.7)	31 (73.8)		
Obese	7 (4.7)		82 (80.4)		
Comorbidity status				34.096	0.025
No comorbidities	3 (6.3)	4 (8.2)	42 (85.7)		
DM	1 (25)	2 (50)	1 (25)		
Hypertension	1 (2.2)	9 (20)	35 (77.8)		
Hypothyroidism	1 (14.3)	3 (42.9)	3 (42.9)		
DM/hypertension	0	6 (18.8)	26 (81.3)		
DM/hypothyroidism	0	2 (100)	0		
Hypertension/hypothyroidism	1 (25)	0	3 (75)		
DM/hypertension/hypothyroidism	0	2 (50)	2 (50)		
Hypertension/seizure	0	0	1 (100)		
Hypertension/DM/hyperthyroid	0	0	1 (100)		
Hypertension/hypothyroid/seizure	0	0	1 (100)		
Charlson’s Comorbidity Index				3.32	0.506
Low: 0–1	3 (4.15)	14 (16.61)	72 (68.23)		
Moderate: 2–3	4 (2.85)	12 (10.27)	39 (42.17)		
High: ≥4	0	2 (1.12)	4 (4.6)		

**Table 4:** Inflammatory aspects, lipid profile, oxygen saturation metrics, and STOP BANG score of patients with different severity groups of OSA.

Variable	AHI	Mean	Median	SD	Q1	Q3	*p*-Value
HB	Mild	12.729	13.4	1.2672	11.4	13.8	0.000
	Moderate	12.736	12.8	1.2673	12.225	13.475	
	Severe	15.448	13.6	18.6356	12.8	14.7	
TC	Mild	8415.71	9050	1046.452	7520	9140	0.192
	Moderate	1008.86	9950	1298.711	9215	10,797.5	
	Severe	9296.92	9620	1305.403	8240	10,210	
Neutrophil	Mild	5.9371	6.5	1.04252	4.8	6.68	0.822
	Moderate	6.598	6.735	0.59281	6.2375	6.9425	
	Severe	6.3721	6.6	0.81509	5.821	7.	
Lymphocytes	Mild	1.9857	2.1	0.33877	1	2.2	0.001
	Moderate	2.1618	2.14	0.44558	1.925	2.5	
	Severe	1.851	1.9	0.38524	1.5	2.12	
Monocyte	Mild	0.19571	0.2	0.007868	0.1	0.2	0.000
	Moderate	0.28029	0.2	0.079018	0.2	0.35	
	Severe	0.43017	0.42	0.08315	0.3	0.54	
Eosinophils	Mild	0.2257	0.24	0.06705	0.1	0.28	0.229
	Moderate	0.3718	0.365	0.13584	0.2	0.5	
	Severe	0.3088	0.29	0.13355	0.21	0.41	
Basophils	Mild	0.02286	0.02	0.013801	0.01	0.04	0.934
	Moderate	0.04357	0.05	0.026835	0.0125	0.0657	
	Severe	0.040	0.04	0.0343	0.02	0.05	
Platelets	Mild	2.3471	2.264	0.226	2.21	2.6	0.668
	Moderate	2.5764	2.425	0.61006	2.0775	3.00075	
	Severe	2.552	2.5	0.503	2.2	2.7	
RDW	Mild	13.671	13.3	1.4637	12.1	14.5	0.043
	Moderate	13.232	13.1	1.262	12.150	14.05	
	Severe	13.673	13.6	0.9091	13	14.2	
CRP	Mild	14.857	11	5.4598	10	11	0.014
	Moderate	12.186	10	6.4937	6.2	10.5	
	Severe	16.289	16	7.3043	10	20	
ESR	Mild	15.57	12	8.619	10	20	0.05
	Moderate	14.79	10	9.118	6.25	10	
	Severe	20.77	22	11.117	10	30	
SIRI	Mild	0.54714	0.6	0.107504	0.43	0.6	0.000
	Moderate	0.85929	0.835	0.221609	0.7225	0.94	
	Severe	1.47396	1.46	0.284	1.28	1.65	
NLR	Mild	3.2	3.091	0.48087	2.8	3.8	0.003
	Moderate	3.1607	3.035	0.67522	2.6725	3.4375	
	Severe	5.018	3.4	16.28	3.1	3.8	
PLR	Mild	119.86	119	17.276	104	128	0.003
	Moderate	124.57	131.5	25.143	100	145.75	
	Severe	140.12	136	29.505	115	162	
TCH	Mild	153.143	154	25.0029	126	164	0.002
	Moderate	149.429	151	25.503	126.5	166.5	
	Severe	166.077	170	29.3006	149	183	
LDL	Mild	94.71	89	18.473	85	110	0.013
	Moderate	88.32	85	30.113	68	109.5	
	Severe	105.38	101	30.881	78	132	
HDL	Mild	37.71	40	6.37	34	42	0.811
	Moderate	37.46	39	5.66	33.25	41	
	Severe	37.57	39	5.012	35	41	
TG	Mild	179.43	169	93.299	135	174	0.047
	Moderate	144.57	134.5	45.563	115.25	175.75	
	Severe	167.17	159	50.938	135	175	
Lowest SPO2	Mild	87.43	91	6.554	80	91	0.000
	Moderate	88.29	90.5	4.008	87	92	
	Severe	80.38	85	11.635	72	89	
Cumulative time duration with SPO2 < 90% (min)	Mild	5.53	6	3.6	3.10	6	0.000
	Moderate	68.60	71.5	32.0	47	73.5	
	Severe	226.2	268.5	85.0	168.9	270.4	
STOP BANG score	Mild	4.42	4	1.122	4	4	0.000
	Moderate	4.714	4.5	1.384	4	4.6	
	Severe	6.5	7	1.32	6.3	7.1	

**Table 5:** Correlation coefficient of significant clinical and biochemical parameters.

Factor	Correlation Coefficient	*p*-Value
Total cholesterol	0.320	0.000
LDL	0.280	0.001
HDL	−0.137	0.096
Triglycerides	0.194	0.017
Lowest SPO2	0.583	0.000
Cumulative time duration with SPO2 < 90%	0.403	0.000
Neck circumference	0.404	0.000
STOP BANG Score	0.347	0.000

**Table 6:** Predictors of the severity of OSA as determined via linear regression for individual inflammatory parameters (standardised ^a^ indicates the standardised intercept in regression analysis).

Model		Unstandardized	Standard Error	Standardized ᵃ	t	*p*	R^2^	F
AHI and SIRI	(Intercept)	−1.262	15.867		−0.080	0.937	0.357	9.231
	AGE	−0.263	0.142	−0.143	−1.853	0.066		
	SEX (M)	8.387	4.041		2.075	0.040		
	BMI	0.544	0.280	0.153	1.940	0.054		
	Total cholesterol	−0.019	0.073	−0.024	−0.262	0.794		
	LDL	0.039	0.071	0.050	0.551	0.582		
	Triglyceride	0.015	0.036	0.034	0.426	0.671		
	CCI	0.261	1.582	0.013	0.165	0.869		
	SIRI	25.848	4.656	0.434	5.552	<0.001		
AHI and Monocyte	(Intercept)	−8.346	16.203		−0.515	0.607	0.348	8.862
	AGE	−0.255	0.143	−0.139	−1.778	0.078		
	SEX (M)	10.061	4.004		2.513	0.013		
	BMI	0.666	0.277	0.187	2.404	0.018		
	Total cholesterol	−0.009	0.074	−0.011	−0.122	0.903		
	LDL	0.025	0.072	0.032	0.352	0.725		
	Triglyceride	0.018	0.036	0.040	0.494	0.622		
	CCI	−0.130	1.591	−0.007	−0.082	0.935		
	Monocyte	90.391	16.952	0.409	5.332	<0.001		
AHI and CRP	(Intercept)	10.021	17.617		0.569	0.570	0.210	4.413
	AGE	−0.307	0.157	−0.167	−1.951	0.053		
	SEX (M)	14.221	4.356		3.265	0.001		
	BMI	0.973	0.302	0.274	3.224	0.002		
	Total cholesterol	−0.029	0.084	−0.035	−0.343	0.732		
	LDL	0.101	0.078	0.128	1.301	0.195		
	Triglyceride	0.019	0.040	0.042	0.470	0.639		
	CCI	−0.275	1.754	−0.014	−0.157	0.876		
	CRP	0.144	0.286	0.043	0.503	0.616		
AHI and ESR	(Intercept)	11.762	17.457		0.674	0.502	0.221	4.719
	AGE	−0.328	0.157	−0.179	−2.090	0.039		
	SEX (M)	14.222	4.284		3.320	0.001		
	BMI	0.917	0.300	0.258	3.054	0.003		
	Total cholesterol	−0.027	0.081	−0.033	−0.329	0.743		
	LDL	0.092	0.077	0.117	1.188	0.237		
	Triglyceride	0.011	0.040	0.025	0.277	0.782		
	CCI	−0.232	1.738	−0.012	−0.133	0.894		
	ESR	0.266	0.180	0.121	1.481	0.141		
AHI and PLR	(Intercept)	−18.672	18.953		−0.985	0.326	0.264	5.975
	AGE	−0.221	0.154	−0.120	−1.436	0.153		
	SEX (M)	13.255	4.177		3.173	0.002		
	BMI	0.908	0.288	0.255	3.152	0.002		
	Total cholesterol	−0.014	0.078	−0.018	−0.182	0.856		
	LDL	0.081	0.075	0.102	1.071	0.286		
	Triglyceride	0.027	0.038	0.062	0.712	0.478		
	CCI	0.160	1.694	0.008	0.094	0.925		
	PLR	0.201	0.063	0.248	3.185	0.002		
AHI and NLR	(Intercept)	−3.618	19.004		−0.190	0.849	0.223	4.779
	AGE	−0.305	0.156	−0.166	−1.957	0.052		
	SEX (M)	13.406	4.330		3.096	0.002		
	BMI	0.944	0.296	0.266	3.185	0.002		
	Total cholesterol	−0.017	0.081	−0.021	−0.213	0.832		
	LDL	0.107	0.077	0.135	1.383	0.169		
	Triglyceride	0.020	0.039	0.045	0.509	0.611		
	CCI	−0.111	1.737	−0.006	−0.064	0.949		
	NLR	4.225	2.636	0.125	1.603	0.111		

**Table 7:** Diagnostic performance of inflammatory markers and STOP-BANG score, alone and in combination, for predicting severe obstructive sleep apnea.

Model	Cut-Off Point	Sensitivity (%)	Specificity (%)	PPV (%)	NPV (%)	Youden’s Index	AUC
A. SIRI	1.105	92.2	91.4	96.1	83	1.84	0.96
B. STOP BANG Score	4.5	86.09	48.6	79.6	60	1.35	0.737
C. SIRI + STOP BANG score + PLR	3.135	98.7	100	100	33.3	0.987	0.987
D. SIRI + STOP BANG Score +PLR + CRP	3.88	100	100	100	100	1	1.00
E. SIRI + STOP BANG score	4	96.6	100	100	16.7	0.96	0.983

## Data Availability

All data generated or analyzed during this study are included in this published article and are available from the corresponding author upon reasonable request.
